# Mammographic image classification with deep fusion learning

**DOI:** 10.1038/s41598-020-71431-x

**Published:** 2020-09-01

**Authors:** Xiangchun Yu, Wei Pang, Qing Xu, Miaomiao Liang

**Affiliations:** 1 School of Information Engineering, Jiangxi University of Science and Technology, Ganzhou, 341000 People’s Republic of China; 2 School of Mathematical and Computer Sciences, Heriot-Watt University, Edinburgh, EH14 4AS UK

**Keywords:** Breast cancer, Applied optics, X-rays

## Abstract

To better address the recognition of abnormalities among mammographic images, in this study we apply the deep fusion learning approach based on Pre-trained models to discover the discriminative patterns between Normal and Tumor categories. We designed a deep fusion learning framework for mammographic image classification. This framework works in two main steps. After obtaining the regions of interest (ROIs) from original dataset, the first step is to train our proposed deep fusion models on those ROI patches which are randomly collected from all ROIs. We proposed the deep fusion model (Model1) to directly fuse the deep features to classify the Normal and Tumor ROI patches. To explore the association among channels of the same block, we propose another deep fusion model (Model2) to integrate the cross-channel deep features using 1 × 1 convolution. The second step is to obtain the final prediction by performing the majority voting on all patches' prediction of one ROI. The experimental results show that Model1 achieves the whole accuracy of 0.8906, recall rate of 0.913, and precision rate of 0.8077 for Tumor class. Accordingly, Model2 achieves the whole accuracy of 0.875, recall rate of 0.9565, and precision rate 0.7,586 for Tumor class. Finally, we open source our Python code at https://github.com/yxchspring/MIAS in order to share our tool with the research community.

## Introduction

Breast cancer is one of the most common types of cancer in women. Early detection and treatment can effectively improve cure rates and reduce mortality. According to the report^1^, early diagnosis and treatment can increase the cure rate of breast cancer from 40 to 90%. Detecting breast cancer using mammographic images is a cost-effective technique, and radiologists can make a diagnosis by analyzing these images. However, the large number of mammographic images produced day by day has brought a huge workload on radiologists and also increased the rate of misdiagnosis. Therefore, developing a computer-aided diagnosis (CAD) system can significantly relieve the pressure on radiologists and improve the diagnosis accuracy. The CAD can assist the radiologists in distinguishing the normal or abnormal tissues and diagnosing the pathological stages. The automatic diagnostic system for mammographic images needs to extract the regions of interest (ROIs) and then classify the ROIs into normal or abnormal (i.e. benign and malignant) tissues. This task is very challenging because the shape and texture information of calcification or masses vary from each other and the presence of blood vessels and muscle fibers also brings interferes to robust recognition^2^. These factors make it very difficult to find competent patterns.

In order to adress this problem, more and more technologies are proposed. Existing research work mainly focuses on feature extraction and classification model selection. Buciu et al.^2^ proposed to extract the Gabor-based features on the patches surrounding the abnormal regions and apply the PCA to conduct dimensionality reduction. Finally, the Proximal Support Vector Machines were utilized to obtain 84.37% whole accuracy in the MIAS dataset. Swiniarski et al.^3^ proposed to extract the 2D Haar wavelet features, then apply the PCA to conduct dimension reduction, and finally utilize the rough set to conduct feature selection. Mammographic images are noisy and have low contrast, which brings a difficulty to well recognizing the calcification or masses. Therefore, Mencattini et al.^4^ proposed a novel algorithm based on dyadic wavelet processing to conduct image denoising and enhancement. Cheng et al.^5^ proposed to extract the descriptors for mammographic image based on Bag-of-Features (BOF) and utilize SVM using normalized histogram intersection to carry out the final classification. Zaiane et al.^6^ focused on the research of classifiers and proposed an association rule-based classifier for mammographic image classification. Zhao et al.^7^ proposed the active learning approach to deal with the problem of limited samples and achieved good accuracy with slightly more labeling cost for mammographic image classification.

Recently, due to the excellent performance of deep learning models in the field of computer vision^8–12^, more and more researchers have begun to study deep learning-based models. Wang et al.^13^ constructed a deep learning model called stacked denoising auto-encoder to classify breast lesions and they obtained satisfactory accuracy by the combined analysis of microcalcifications and breast masses. Huynh et al.^14^ proposed to use the deep models based on transfer learning to extract the deep features for breast lesions and achieved good performance compared with the analytically extracted features. Li et al.^15^ applied the convolutional neural networks (CNN) to recognize the abnormalities and achieved a high sensitivity for benign or malignant classification. Lévy et al.^16^ adopted the transfer learning approach to classify the collected breast masses and obtained satisfactory results. Cai et al.^17^ proposed a CNN model to conduct breast microcalcification diagnosis. In addition, in order to make full use of the advantages of handcrafted features, in that research they fused handcrafted features and deep features to improve the performance of the model.

**Figure 1:**
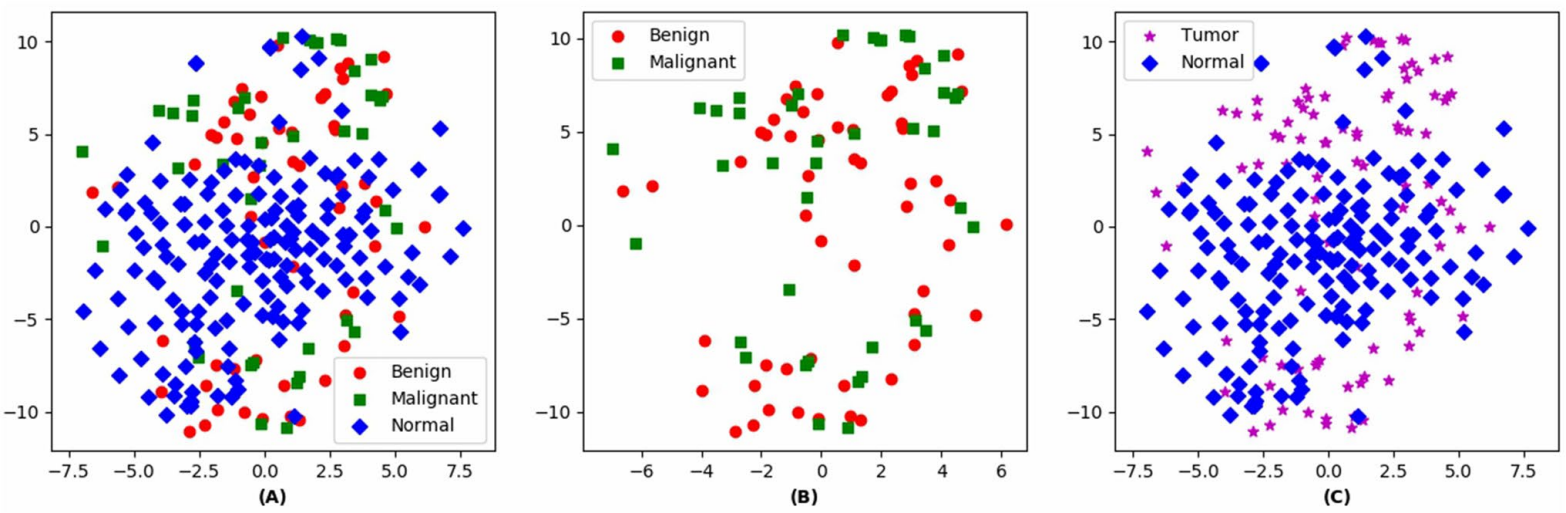
The ROI images visualization using t-SNE. (**A**) Visualization for three classes (i.e. Benign, Malignant, and Normal classes). (**B**) Visualization for Benign and Malignant classes. (**C**) Visualization for Tumor (Benign and Malignant) and Normal classes.

In this research, we explore to utilize the deep fusion models to perform the mammographic image classification in the MIAS dataset^18^. First, the images in MIAS are preprocessed to remove noise and enhance image quality. Then, the ROIs of abnormal class (i.e. benign and malignant) are collected, and those derived from the Normal class are extracted from random locations. In the MIAS dataset, the specific center coordinate for each abnormal region is annotated, so one square area with this center coordinate is extracted as the ROI. No specific location information is given for Normal class, therefore a square area of the above size is randomly extracted from the whole image as the ROI. As shown in Fig. 1, we have visualized the samples from the training set using t-SNE^19^. And we find that the benign and malignant ROIs share similar patterns and lack distinguishability from each other, while the Normal class and Tumor (i.e. abnormal) class have a certain degree of distinguishability. Therefore, we propose to design deep fusion models to discover patterns that are distinguishable between Normal and Tumor categories.

**Figure 2:**
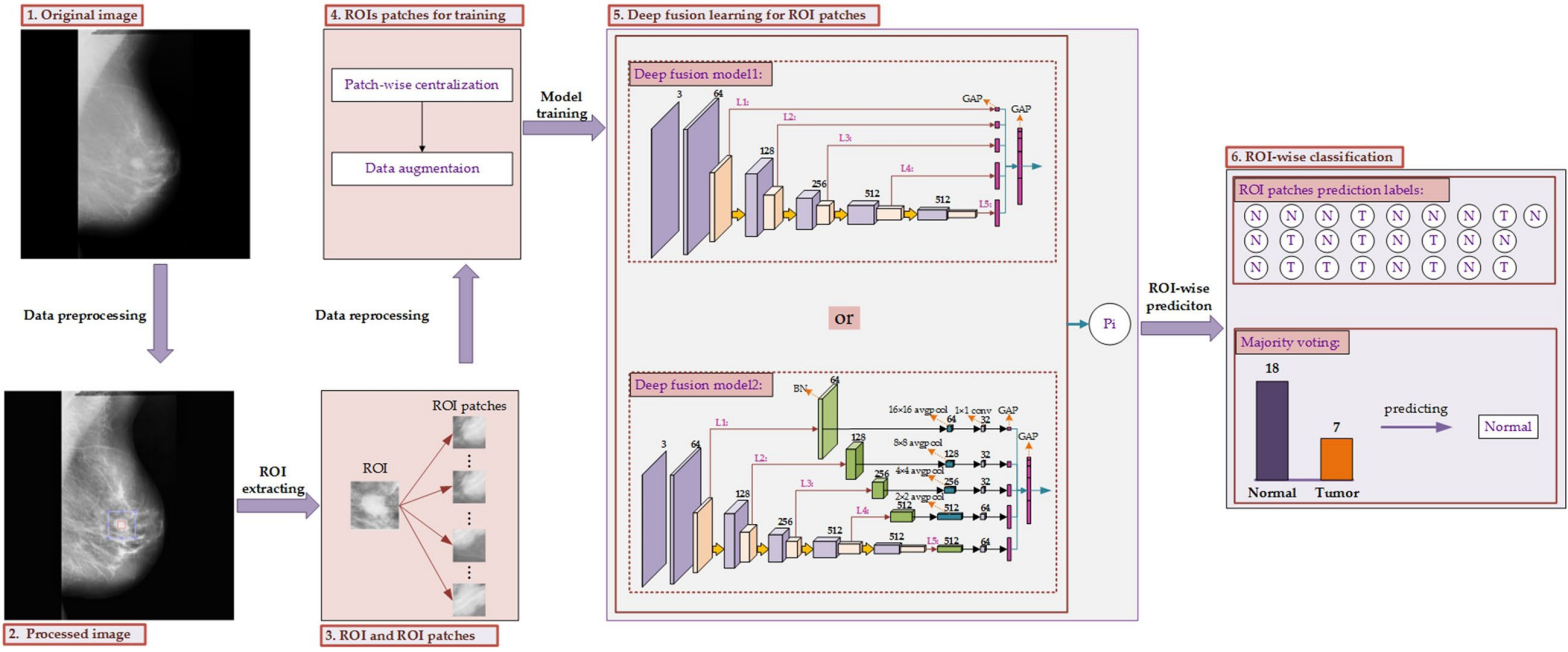
The whole framework for deep fusion learning. First, the original image (1) is converted to the processed image (2) by data preprocessing with an aim to remove noise and conduct image enhancement. Then the region of interest (ROI) is extracted and the corresponding patches are collected from it by random patch sampling. Finally, we will obtain the ROI and ROI patches (3) from the target image. Besides, the ROI patches need to be reprocessed by conducting patch-wise centralization and data augmentation to obtain adjusted ROI patches for training (4). Then, the deep fusion learning (5) is conducted among those ROI patches including two kinds of deep fusion models. Finally, the ROI-wise classification (6) is carried out using majority voting to obtain the final prediction.

It is not feasible to train a deep fusion model on such a small sample set. Besides, the shape and texture information of abnormities are difficult to extract among the ROIs. Therefore, we proposed to train our deep fusion models on the ROIs patches which are randomly extracted from the ROIs. Finally, the majority voting is employed to calculate the predictions of all patches for one whole ROI to achieve the final prediction. We have proposed two different deep fusion models, one is to directly extract deep features derived from each block, and the other to further explore the cross-channel deep patterns among each block. The whole framework for deep fusion learning in this research is presented in Fig. 2.

## Materials and methods

### Datasets

**Figure 3:**
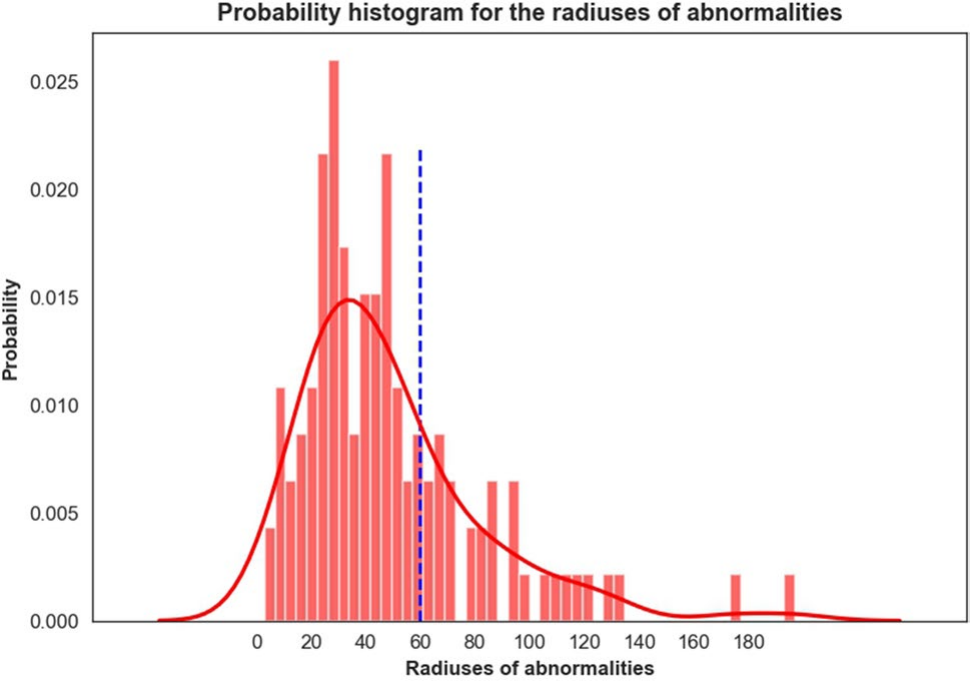
The data distribution of all radiuses of abnormities.

The mammogram images used in this research are collected from the MIAS dataset^18^. The dataset consists of two categories, normal class and abnormal class (i.e. benign and malignant). There are 208 normal images, 114 abnormal images including 63 benign images and 51 malignant images. Each image is 1,024 × 1,024 pixels. Each abnormal region is annotated with center coordinate and approximate radius. The data distribution of all radiuses is shown in Fig. 3. The size of radiuses ranges from 3 to 197 and most of them are around 40. In order to better capture the global shape information of each abnormal region, we choose to extract ROI which can cover the annotated region with radius of 60 (blue dotted line in Fig. 3). It can ensure no less than 70% of annotated regions covered. Finally, these square areas with the annotated center coordinates whose side length is 120 (60 × 2) are extracted as the ROIs for abnormal class. No specific center coordinates are given for normal class, therefore these square area of the above size are randomly extracted from the whole image as the ROIs for Normal class.

### Data preprocessing

**Figure 4:**
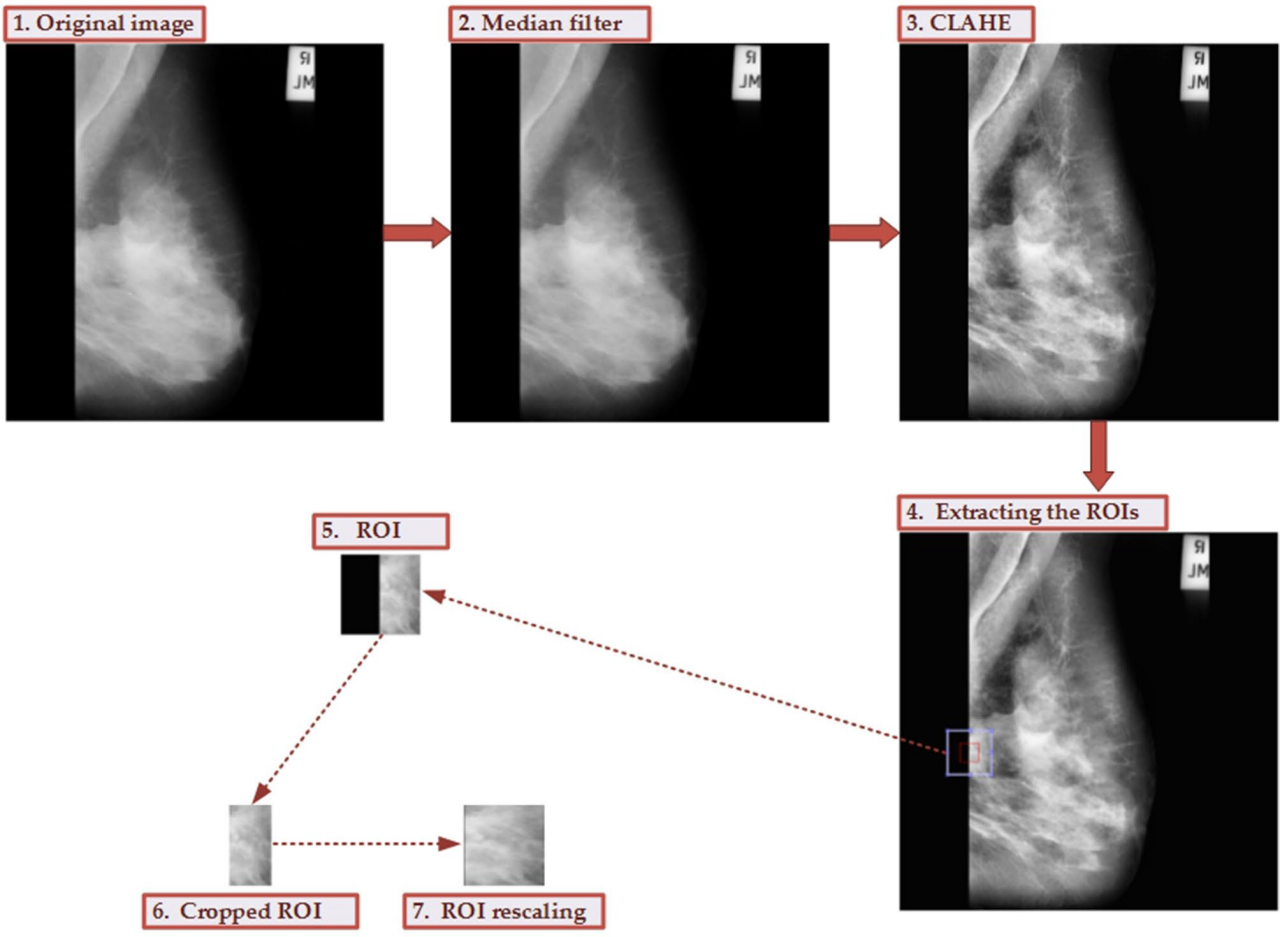
The whole process for data preprocessing and ROI extraction. The noise from the original image (1) is removed using the median filter (2). Then the image enhancement is performed using CLAHE (3). Extracting the ROIs (4) is carried out to obtain the ROI marked using a light blue box (the red box denotes the originally labeled area). And the black part of ROI (5) is removed to obtain the cropped ROI (6). Finally, the ROI rescaling is carried out to achieve the final ROI (7).

The original images from the MIAS dataset have strong noise. In order to remove the noise and improve image quality, the data preprocessing is essential before conducting model learning. The flowchart for the data preprocessing is given in Fig. 4. The median filter is employed to remove the noise, contrast limited adaptive histogram equalization (CLAHE)^20^ is used to perform image enhancement. After extracting the ROIs, the non-breast area is removed and is rescaled to 120 × 120 pixels. We provide the corresponding MATLAB scripts for this data preprocessing, and the source codes are available at https://github.com/yxchspring/MIAS_Preprocess.

After data preprocessing, we obtain the final ROIs with 120 × 120 pixels surrounding the centers of 114 abnormal regions. For normal images, we extract the corresponding ROIs with 120 × 120 pixels at the random center within the breast area. Finally, we collected 207 normal ROIs and 119 abnormal ROIs (i.e. 68 benign, 51 malignant). We divide the data into three parts, 60% as the training set, 20% as the validation set, and the remaining 20% as the testing set.

### ROI patches extraction

After we have obtained the 207 normal ROIs and 119 abnormal ROIs, we randomly extract the small patches of 72 × 72 pixels on each ROI. In this research, we collect 500 small patches for each normal ROI and 2,000 small patches for each abnormal ROI. According to the data splitting of the ROIs, we collect 206,697 ROI patches for the training set, 68,565 ROI patches for the validation set, and 66,564 ROI patches for the testing set. These training and validation data are used to train our proposed deep fusion models, and finally the majority voting is adopted to carry out the ROI-wise classification by integrating the predictions from ROI patches of testing set.

## Methods

**Figure 5:**
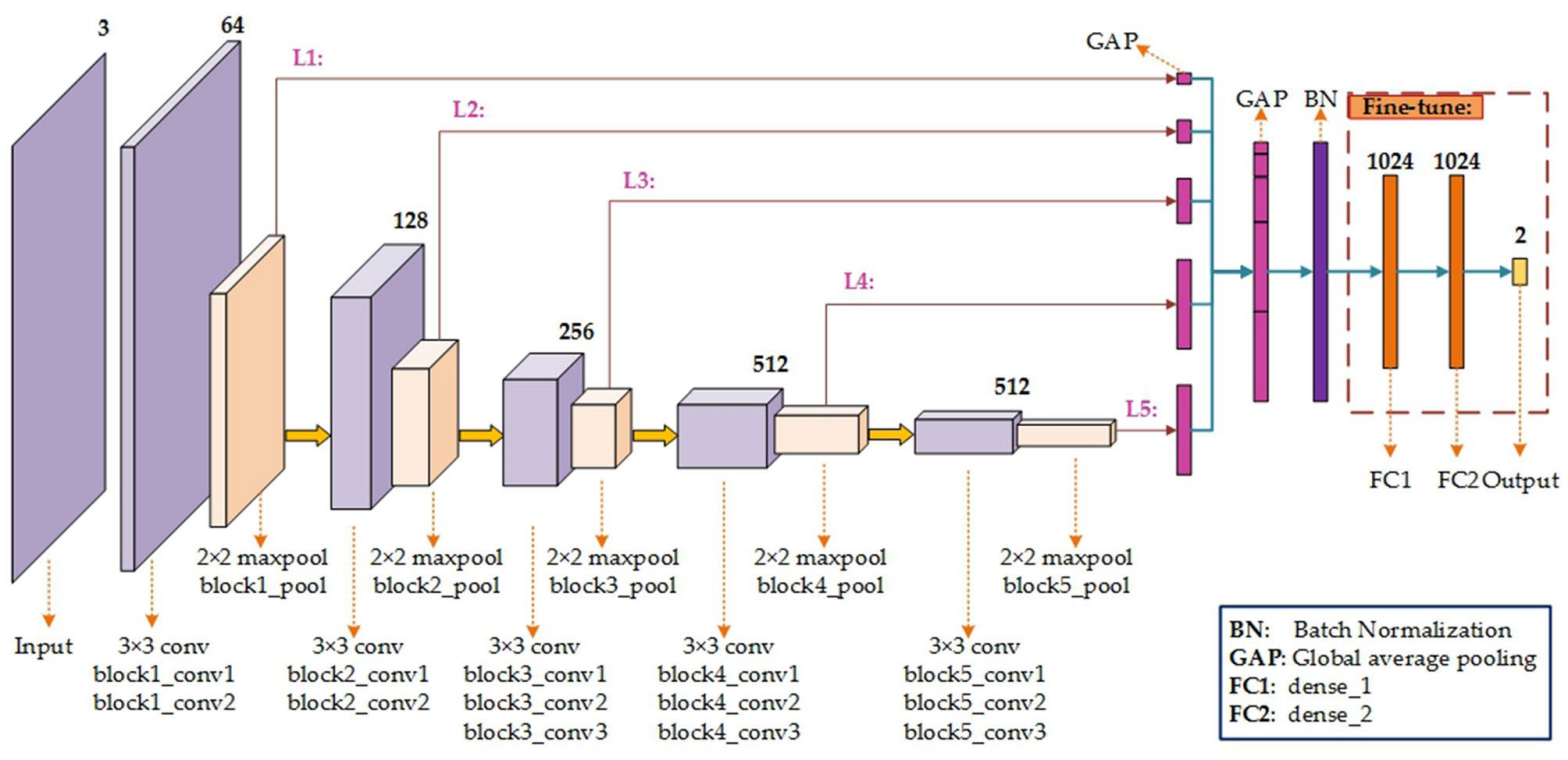
The network structure of Model1 based on VGG16^21^. The GAP layer is added into each branch to obtain the global information for each block, respectively. Then, the deep features derived from L1–L5 branches are concatenated into a longer GAP layer, and this is followed by the BN, FC1, FC2, and Output layers. During model training, the weights in the red dotted box are fine-tuned to learn the domain-specific knowledge.

In this section, we propose two deep fusion models, Model1 (e.g. VGG16_Fusion1) and Model2 (e.g. VGG16_Fusion2). Figure 5 depicts the specific model structure of VGG16_Fusion1 based on the pre-trained VGG16. And the model structure based on VGG19 is similar. The pre-trained VGG16^21^ has five blocks and each maxpool layer covers the different ‘granularity’ of deep information. The shallow layer captures the local patterns (e.g. edges) and the deep layer will capture global patterns (e.g. organization structure). Fusing all the five layers (L1–L5 as shown in Fig. 5) will enrich the characterization information of the input data and improve the discrimination of the classification model. The specific approach is to conduct the global average pooling (GAP) for each pooling layer of each block and concatenate them to form the longer GAP layer. The GAP layer will be connected to the ‘BN’ layer (Batch Normalization) followed by the FC1 (Fully Connected), FC2, and Output layers. The weights of the ‘conv’ layers will be initialized by the pre-trained VGG16, and the weights of the remaining layers are randomly initialized. While the weights of ‘conv’ layers are frozen during model training, the weights of FC1, FC2, and Output layers are fine-tuned to learn the domain-specific knowledge.

The information between different channels will be highly correlated. The 1 × 1 convolution^22,23^ will integrate the cross-channel information and further achieve dimensionality reduction, and can effectively reduce the model parameters. Therefore, we propose the second deep fusion model, Model2 (VGG16_Fusion2).

**Figure 6:**
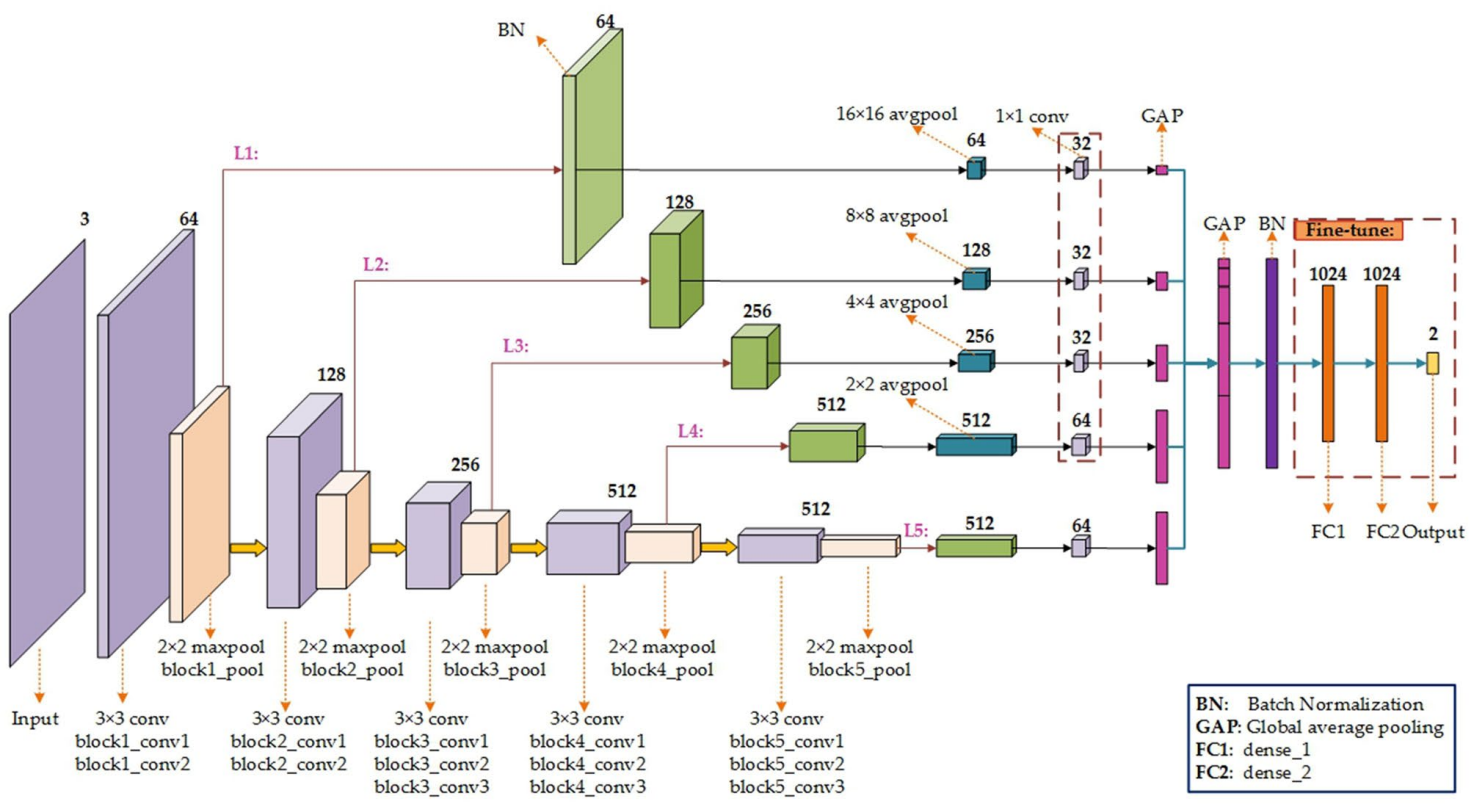
The network structure of Model2 based on VGG16^21^. The BN, avgpool, 1 × 1 conv, and GAP layers are added into L1-L5 branches, respectively. Then, the deep features derived from L1-L5 branches are concatenated into a longer GAP layer, and followed by the BN, FC1, FC2, and Output layers. During model training, the weights in the red dotted box are fine-tuned to learn the domain-specific knowledge.

For L1-L5 branches, the specific parameters setup of ‘BN’, ‘avgpool’, and ‘1 × 1 conv’ layers are shown in Fig. 6. Then, the GAP information derived from each ‘1 × 1 conv’ layer will be concatenated to form the longer GAP layer. The subsequent operations are similar to VGG16_Fusion1. It is worth noting that the weights of the ‘conv’ layers will be initialized by the pre-trained VGG16, and the ones of the remaining layers including the five branches are randomly initialized. While the weights of the ‘conv’ layers are frozen during model training, the weights of ‘1 × 1 conv’ layers of five branches, FC1, FC2, and Output layers are fine-tuned to learn the domain-specific knowledge. The specific model structure is shown in Fig. 6.

When the predictions of all patches extracted from one ROI *i* are made, we can get the final prediction for ROI *i* by the majority voting strategy as shown below:

1 $$\widehat{{y}_{i}}=mode\{{l}_{1}\left({x}_{i}\right),{l}_{2}\left({x}_{i}\right),\cdots ,{l}_{j}\left({x}_{i}\right),{\cdots ,l}_{n}\left({x}_{i}\right)\}$$

where *x*_*i*_ denotes the ROI *i*, *l*_*j*_*(x*_*i*_*)* denotes the prediction label of the *j-th* patch of ROI *i*, *n* is the total number of patches of ROI *i*, and *n* is set to be 25. The *mode* function can obtain the mode (i.e. majority) of the prediction labels of all patches from one whole ROI *i*.

## Results

### Experimental results for mammographic ROI-wise classification

**Figure 7:**
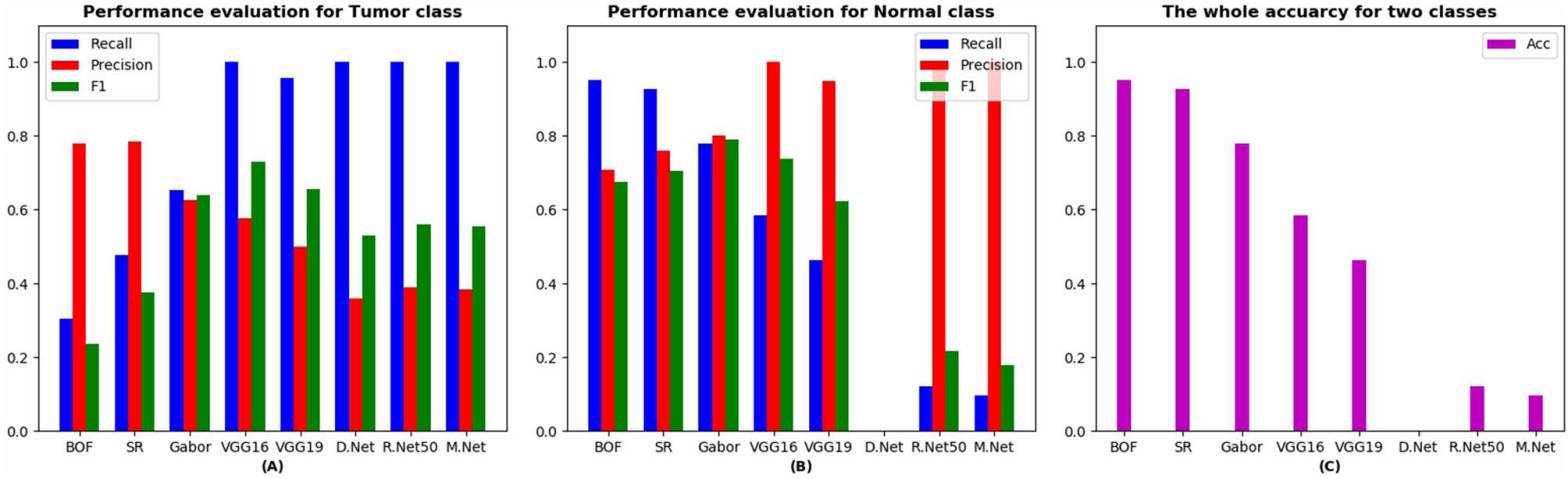
The performance evaluation for all comparative experiments. (**A**) Evaluation results for Tumor class. (**B**) Evaluation results for Normal class. (**C**) The whole accuracy for Tumor and Normal classes.

Table 1 shows the experimental results for comparison models, including Bag-of-Features (BOF^24,25^), Sparse Representation (SR^26,27^), Gabor features^28,29^, and deep models, including VGG16^21^, VGG19^21^, DenseNet^30^, ResNet50^31^, and MobileNet^32^. From Table 1 we can see that although the comparison models obtain good whole accuracy rate (Acc), they achieve a low recall rate for Tumor (T) class. However, to obtain a high recall rate is necessary for medical image classification. We find that all the deep models achieve a high recall rate. But the DenseNet, ResNet50, and MobileNet have not achieved good results in terms of recall rate, precision rate, and whole accuracy rate for Normal (N) class. Only the VGG16 and VGG19 have achieved good results, so we will present the experimental results of our proposed deep fusion models based on VGG16 and VGG19. Figure 7 presents a more intuitive comparison of experimental results.

**Table 1:** The performance for the pre-trained models.

Model	Class	Recall	Precision	F1	Acc
BOF	N	0.9512	0.7091	0.6745	0.7188
	T	0.3043	0.7778	0.2367	
SR	N	0.9268	0.7600	0.7044	0.7656
	T	0.4783	0.7857	0.3758	
Gabor	N	0.7805	0.8	0.7901	0.7344
	T	0.6522	0.625	0.6383	
VGG16	N	0.5854	1	0.7385	0.7344
	T	1	0.5750	0.7302	
VGG19	N	0.4634	0.9500	0.6230	0.6406
	T	0.9565	0.5000	0.6567	
DenseNet	N	0	0	0	0.3594
	T	1	0.3594	0.5287	
ResNet50	N	0.1220	1	0.2174	0.4375
	T	1	0.3898	0.5610	
MobileNet	N	0.0976	1	0.1778	0.4219
	T	1	0.3833	0.5542

Table 2 presents the experimental results of our proposed deep fusion model1, VGG16_Fusion1, and VGG19_Fuion1. It can be discerned from Table 2 that as the number of fusing branches increases, the whole accuracy rate ordinarily increases. In theory, fusing more information will enhance classification performance. For VGG16_Fusion1, the VGG16_Fusion1(1–5) obtains the best result. For VGG19_Fusion1, VGG16_Fusion1(2–5) obtains the best results. VGG16_Fusion1(1–5) performs well on all the evaluation metrics, especially for Tumor class. VGG19_Fusion1(2–5) and VGG19_Fusion1(1–5) both achieve good results on all the evaluation metrics and VGG19_Fusion1(2–5) achieves the better whole accuracy rate, while VGG19_Fusion1(1–5) achieves the better recall rate for Tumor class.

**Table 2:** The experimental results for Model1.

Model	Branch	Class	Recall	Precision	F1	Acc
VGG16_Fusion1	[4, 5]	N	0.6341	1	0.7761	0.7656
		T	1	0.6053	0.7541	
	[3, 4, 5]	N	0.7805	0.8649	0.8205	0.7812
		T	0.7826	0.6667	0.7200	
	[2, 3, 4, 5]	N	0.8537	0.8974	0.8750	0.8438
		T	0.8261	0.7600	0.7917	
	[1, 2, 3, 4, 5]	N	0.8780	0.9474	0.9114	0.8906
		T	0.9130	0.8077	0.8571	
VGG19_Fusion1	[4, 5]	N	0.7317	0.9677	0.8333	0.8125
		T	0.9565	0.6667	0.7857	
	[3, 4, 5]	N	0.8537	0.8974	0.8750	0.8438
		T	0.8261	0.7600	0.7917	
	[2, 3, 4, 5]	N	0.9268	0.9048	0.9157	0.8906
		T	0.8261	0.8636	0.8444	
	[1, 2, 3, 4, 5]	N	0.8780	0.9231	0.9000	0.8750
		T	0.8696	0.8000	0.8333	

**Figure 8:**
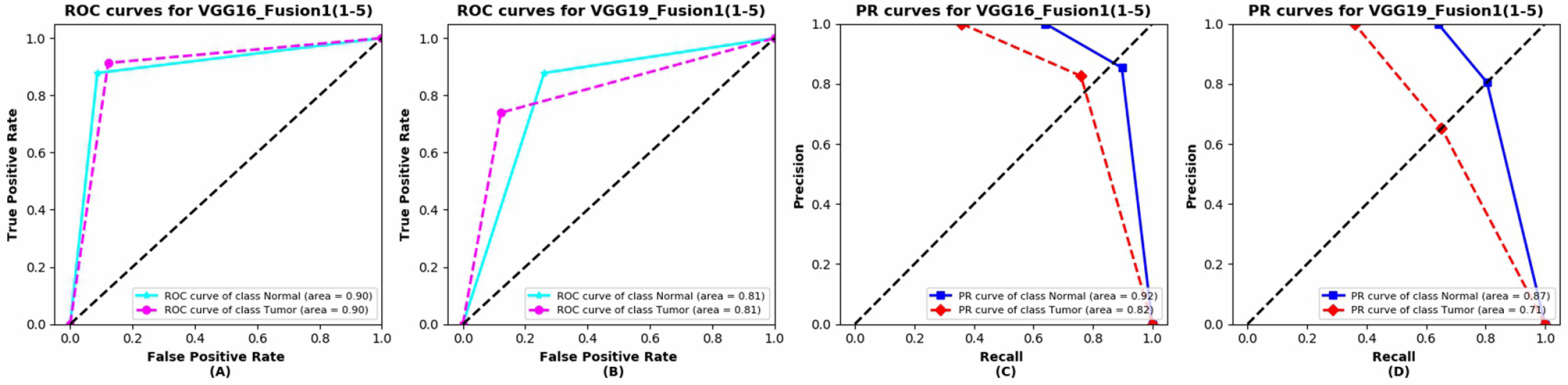
The ROC curves and Precision-Recall (PR) curves of VGG16_Fusion1(1–5) and VGG19_Fusion1(1–5). (**A**) ROC curves for VGG16_Fusion1(1–5). (**B**) ROC curves for VGG19_Fusion1(1–5). (**C**) PR curves for VGG16_Fusion1(1–5). (**D**) PR curves for VGG16_Fusion1(1–5).

The ROC curves and Precision-Recall (PR) curves of VGG16_Fusion1(1–5) and VGG19_Fusion1(1–5) are shown in Fig. 8. The two kinds of models both achieve a good area under curve (AUC) of ROC and PR curves. The VGG16_Fusion1(1–5) obtains better performance compared with VGG19_Fusion1(1–5).

Table 3 presents the experimental results of our proposed deep fusion model2, VGG16_Fusion2, and VGG19_Fusion2. The 1 × 1 convolution will discover the cross-channel deep information. Branch 1 will explore the cross-channel local patterns, the subsequent branches will explore the more ‘abstract’ cross-channel patterns (e.g. the structure-scale patterns). The VGG16_Fusion2 obtains the better recall and whole accuracy rates compared with VGG16_Fusion1, while the whole accuracy of VGG16_Fusion2(1–5) is slightly lower than VGG16_Fusion1(1–5). This can well validate that the cross-channel patterns and therefore improve classification performance. Compared with VGG19_Fusion1, VGG19_Fusion2 does not perform well in terms of the whole accuracy rate but obtains a better recall rate for Tumor class, which is consistent with the goal of medical image classification.

**Table 3:** The experimental results for the Model2.

Model	Branch	Class	Recall	Precision	F1	Acc
VGG16_Fusion2	[5]	N	0.4878	1	0.6557	0.6719
		T	1	0.5227	0.6866	
	[4, 5]	N	0.7561	0.9118	0.8267	0.7969
		T	0.8696	0.6667	0.7547	
	[3, 4, 5]	N	0.8049	0.9429	0.8684	0.8438
		T	0.9130	0.7241	0.8077	
	[2, 3, 4, 5]	N	0.7805	0.9697	0.8649	0.8438
		T	0.9565	0.7097	0.8148	
	[1, 2, 3, 4, 5]	N	0.8293	0.9714	0.8947	0.8750
		T	0.9565	0.7586	0.8462	
VGG19_Fusion2	[5]	N	0.4634	1	0.6333	0.6562
		T	1	0.5111	0.6765	
	[4, 5]	N	0.6585	0.9000	0.7606	0.7344
		T	0.8696	0.5882	0.7018	
	[3, 4, 5]	N	0.6829	0.9655	0.8000	0.7812
		T	0.9565	0.6286	0.7586	
	[2, 3, 4, 5]	N	0.7317	0.9375	0.8219	0.7969
		T	0.9130	0.6562	0.7636	
	[1, 2, 3, 4, 5]	N	0.7561	1	0.8611	0.8438
		T	1	0.6970	0.8214	

**Figure 9:**
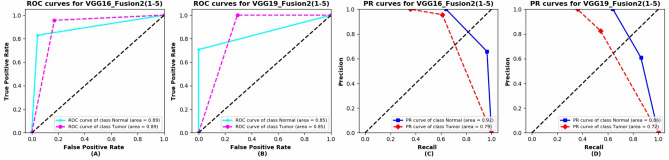
The ROC and PR curves of VGG16_Fusion2(1–5) and VGG19_Fusion2(1–5). (A) ROC curves for VGG16_Fusion2(1–5). (**B**) ROC curves for VGG19_Fusion2(1–5). (**C**) PR curves for VGG16_Fusion2(1–5). (**D**) PR curves for VGG16_Fusion2(1–5).

The ROC and PR curves of VGG16_Fusion2(1–5) and VGG19_Fusion2(1–5) are presented in Fig. 9. The VGG16_Fusion2(1–5) obtains better performance compared with VGG19_Fusion2(1–5). This may reveal that when the model goes deeper, the overfitting issues will occur and the generalization ability for medical image tasks may decline. And the local patterns may play a more important role concerning those tasks. This may explain why DenseNet, ResNet50, and MobileNet do not perform well in this task.

**Figure 10:**
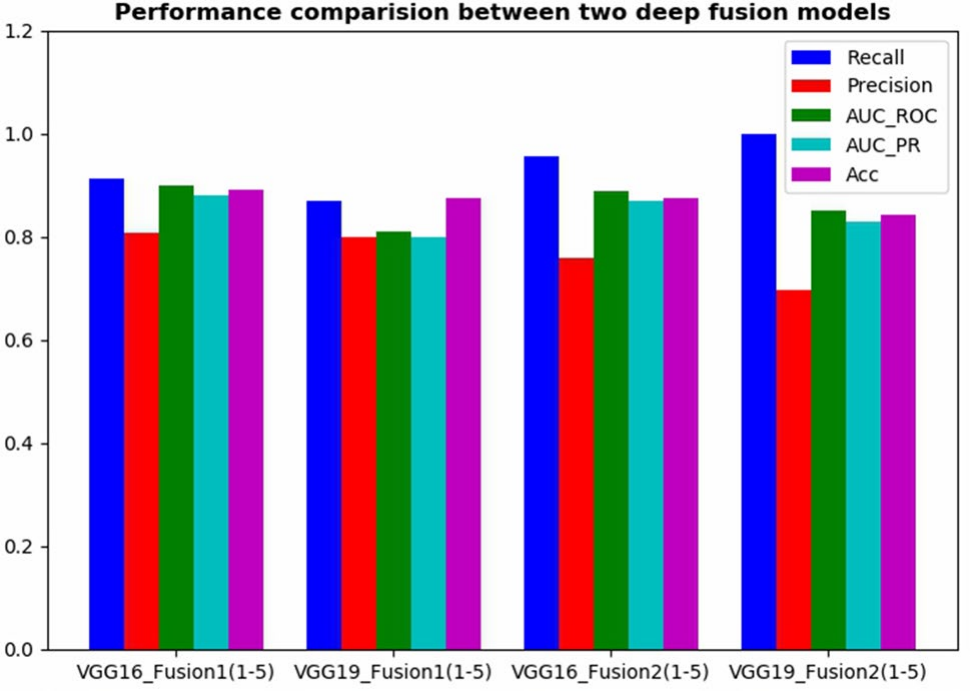
The performance comparison between Model1 and Model2.

Figure 10 presents recall, precision, AUC of ROC curve, AUC of PR curve of Tumor class, and the whole accuracy rates for VGG16_Fusion1(1–5), VGG19_Fusion1(1–5), VGG16_Fusion2(1–5), and VGG19_Fusion2(1–5). The Model2 models obtain a higher recall rate while Model1 models obtain a higher precision rate. To pursue a higher recall rate, Model2 will be more competent for our particular medical image classification task to some extent.

## Conclusion

In this research, we proposed a deep fusion learning framework for mammographic image classification. In order to address the interference derived from various shape and texture information among those calcification or masses,we divide this task into two steps. The first step is to extract a large number of small patches from the original ROIs set, and then train a deep fusion model on these small ROIs patches set. It can contribute to mitigating the overfitting issues when conducting deep model training. The second step is to integrate the prediction results obtained in the first step. The majority voting is used to carry out the ROI-wise classification.

Besides, we propose two deep fusion models, Model1 and Model2. Model1 can directly fuse deep information covering multiple scales, thereby improving the model discriminability. Model2 further explores the cross-channel deep features and the experimental results show that this can improve the recall rate of the Tumor class. Our follow-up work is to further explore the deep fusion learning that can distinguish the contribution of different branches. We believe that the model robustness can be enhanced by fusing different levels of patterns with different weights.

## Data Availability

The mammographic image data used to support the findings of this study are openly available from the MAMMOGRAPHIC IMAGE ANALYSIS SOCIETY (https://peipa.essex.ac.uk/pix/mias/all-mias.tar.gz).
